# Intravenous Reelin rescues despair-like behavior, Reelin cells in the dentate sub-granular zone, and spleen atrophy in the cyclic corticosterone model of recurring depressive episodes

**DOI:** 10.3389/fphar.2024.1368620

**Published:** 2024-02-23

**Authors:** B. S. Reive, J. Johnston, C. L. Sánchez-Lafuente, Kaylene Scheil, K. Kurz, L. E. Kalynchuk, H. J. Caruncho

**Affiliations:** ^1^ Division of Medical Sciences, University of Victoria, Victoria, BC, Canada; ^2^ Department of Biology, University of Victoria, Victoria, BC, Canada

**Keywords:** antidepressant, chronic stress, Reelin, major depression, rodents

## Abstract

Novel antidepressants are predominantly evaluated preclinically in rodent models of chronic stress in which animals experience a single prolonged exposure to chronic stress prior to treatment. Rodent models of a single episode of chronic stress translate poorly to human depressive disorders, which are commonly marked by recurring depressive episodes. Intravenous administration of Reelin has previously been shown to resolve immobility in the forced swim test of rats exposed to a single prolonged exposure to chronic stress. To determine whether Reelin has antidepressant-like properties in a model of recurring depressive episodes, Long–Evans rats (N = 57) were exposed to multiple cycles of chronic stress and stress-free periods before the administration of a single injection of Reelin during the final cycle of chronic stress. The animals then performed in the forced swim test and open field test before the post-mortem evaluation of Reelin cell counts in the sub-granular zone of the dentate gyrus to determine the impact of treatment on hippocampal Reelin levels and spleen white pulp to evaluate the role of Reelin treatment in peripheral inflammation. The results show a single Reelin injection reversed elevated levels of immobility in the forced swim test in both male and female subjects exposed to the cyclic chronic stress model of recurring depressive episodes. Treatment with Reelin also restored Reelin-positive cell counts in the dentate gyrus sub-granular zone and reversed atrophy of spleen white pulp. The results shown here indicate that treatment with Reelin could effectively resolve alterations in forced swim test behavior caused by the cyclic corticosterone model of recurring depressive episodes and that Reelin homeostasis is important for regulating stress-related inflammation. Future preclinical antidepressant research should incorporate models of multiple depressive episodes to improve the translation of preclinical rodent research to human depressive disorders.

## Introduction

Major depression is a leading cause of disability globally (Rehm and Shield, 2019). Stress is perhaps the most common precipitant of depressive episodes in humans, which occur following any number of stressors (or combinations of stressors), such as social, psychological, economic, or physiological stress. Normal human life often involves periods of considerable stress, followed by periods of relative calm, and while some individuals are resistant to stress, others repeatedly experience depressive episodes. For many people depressive episodes are self-remitting, but relapse remains a concern long after depressive symptoms resolve because the history of depression impacts the susceptibility to develop depressive symptoms upon future exposure to stress (Shapero et al., 2013; Technow et al., 2015). Additionally, history of depression can impact resistance to antidepressant treatment and has been linked to various long-term health concerns including, but not limited to, immune dysfunction, increased risk of heart disease, and dementia later in life (Blume et al., 2011; Carney and Freedland, 2016; Song et al., 2020; Saiz-Vazquez et al., 2021; Richmond-Rakerd et al., 2022).

Most rodent research evaluating antidepressant treatment efficacy is conducted in rodent models involving prolonged exposure to stress with no intermittent recovery and self-remittance, such as in models of chronic unpredictable mild stress, chronic restraint stress, chronic social instability, and chronic corticosterone (CORT) exposure (Kalynchuk et al., 2004; Marks et al., 2009; Herzog et al., 2009; Chiba et al., 2012; Antoniuk et al., 2019). This translates poorly to the human condition often marked by a lengthy history of depressive episodes. To resolve this limitation, a cyclic chronic stress rodent model was developed involving multiple bouts of chronic stress and intermittent stress-free periods, during which depression-like behavior self-remits, followed by additional exposure to chronic stress (Lebedeva et al., 2020). In the cyclic model, rodents develop depression-like behavior during the second and third cycles with fewer exposures to the stressor and depression-like behavior persists longer into the recovery period relative to those only exposed to a single prolonged period of chronic stress (Lebedeva et al., 2020). However, antidepressant efficacy is yet to be evaluated within the cyclic CORT model of recurring depressive episodes, which is required to validate the use of this model.

Reelin, an extracellular matrix protein important for the lamination of the brain during development, regulates synaptic plasticity in the adult brain (reviewed by Faini et al., 2021; reviewed by Weeber et al., 2002). Reelin is a large protein (∼410 kDa) expressed in the brain and numerous peripheral organs regulating various processes involved in cell survival, cell proliferation, and cellular adhesion (as reviewed by Carpenter and Freedland, 2016; reviewed by Alexander et al., 2023). Post-mortem brain samples from individuals diagnosed with major depression, bipolar disorder, and schizophrenia were shown to express a reduced number of Reelin-positive cells in the hippocampus (Fatemi et al., 2000). Although the functional impact of reduced Reelin expression is poorly understood in humans, research shows that the depletion of Reelin renders rodents susceptible to developing depressive symptoms, following chronic stress (Lussier et al., 2011). Recently, our laboratory showed antidepressant-like effects of recombinant Reelin treatment in rats exposed to chronic stress, through either intraventricular or intravenous administration (Brymer et al., 2020; Allen et al., 2022; Johnston et al., 2023). Treatment with Reelin also resolves various neurotransmitter abnormalities associated with exposure to chronic stress, stress-induced spatial memory deficits, and long-term potentiation (LTP) (Brymer et al., 2020; Allen et al., 2022; Johnston et al., 2023). Although the mechanisms through which Reelin regulates neuronal function to alleviate depression-like behavior in chronically stressed rats is not fully elucidated, regulating synaptic plasticity is one key mechanism through which Reelin treatment reverses stress-induced depression-like behavior, as AMPA receptor blockade prevented the antidepressant-like effects of Reelin treatment in rodents exposed to chronic stress (Brymer et al., 2020).

To address whether a single injection of Reelin maintains antidepressant-like effects in animals exposed to multiple bouts of chronic stress, rats were exposed to either 1.3 or 2.6 “cycles” of repeated CORT injections before receiving either Reelin (3 ug) or a vehicle through tail vein injections on the final day of stress exposure. The animals then performed in the forced swim test (FST) to evaluate potential antidepressant-like effects of Reelin and the open field test (OFT) to evaluate anxiety-like behavior and differences to locomotion. We then evaluated Reelin-positive cell counts in the dentate gyrus (DG) and gross morphology of the spleen to determine whether Reelin treatment reversed Reelin depletion in the hippocampus and/or resolved white pulp atrophy in animals exposed to multiple cycles of chronic stress.

## Methods

### Animals

Long–Evans rats (N = 57) aged approximately 12–15 weeks and weighing approximately 200–400 g at the start of the experiment were used in the experiment (18 males and 39 females). The rats in the 2.6-cycle cohort were approximately 12 weeks of age at the start of the experiment and included 18 males and 18 females, and the rats in the cohort receiving 1.3 cycles of CORT were approximately 15 weeks of age, which included 21 female rats. The rats were bred in the University of Victoria Animal Care Facility and were single-housed in clear plastic cages in a colony room maintained at a constant temperature of 22 ±1°C and a 12:12 h light/dark cycle (lights on at 07:00 h and off at 19:00 h) for the duration of the study. As CORT-treated rats, primarily males, can become more territorial and defensive during chronic CORT exposure, leading to cage conflicts, we ensured that all rats, irrespective of the condition or sex, were single-housed throughout the experiment for consistency. The rats were briefly handled once daily for 7 days prior to the experiment. All experimental procedures were carried out during the light period of the light/dark cycle and were conducted in accordance with the regulations outlined by the University of Victoria Committee on Animal Care and the Canadian Council on Animal Care.

### Cyclic CORT model

**FIGURE 1:**
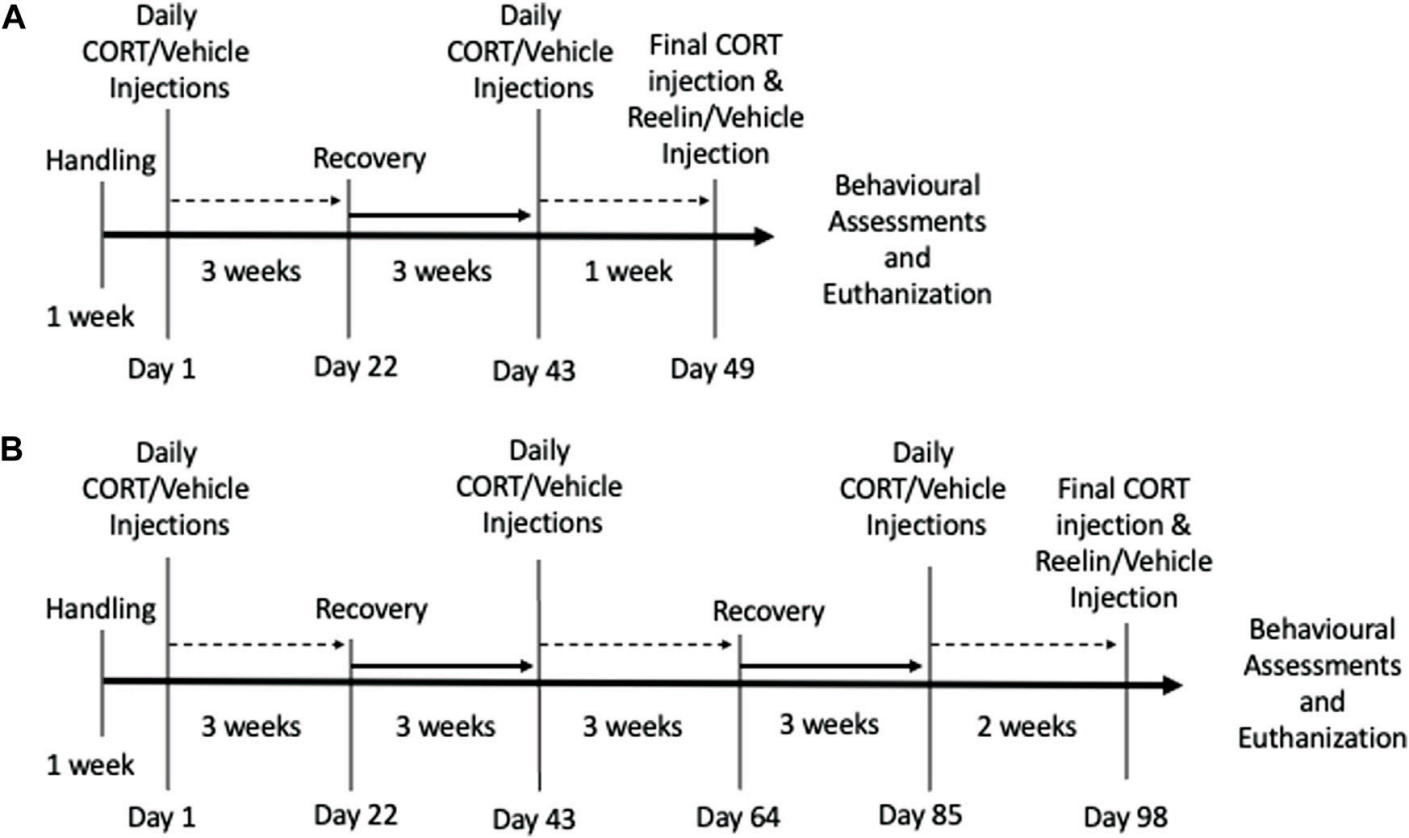
Experimental timeline of 1.3 cycles and 2.6 cycles. **(A)** Experimental timeline of animals exposed to 1.3 cycles of chronic stress. **(B)** Experimental timeline of animals exposed to 2.6 cycles of chronic stress.

The experimental animals were randomly assigned to receive chronic CORT subcutaneous injections at a dose of 20 mg/kg (1 mL/kg, s.c.; Steraloids, Newport, RI) or vehicle injections. CORT was suspended in 0.9% (w/v) physiological saline (pH = 7.4) and 2% (v/v) Tween-80 (Sigma-Aldrich, Germany). Injections were administered for 21 days, followed by a 21-day injection-free recovery period, which represented one cycle of CORT. One cohort of female rats was exposed to a total of 1.3 cycles, or a total of 28 injections, and a cohort of males and females was exposed to 2.6 cycles of CORT for a total of 56 injections. The main focus is to evaluate antidepressant-like effects of Reelin after multiple periods of chronic stress; therefore, we evaluated both sexes in the cohort exposed to 2.6 cycles of CORT, where deficits were expected to be more severe. As depression predominantly affects females, females were selected for the cohort limited to 1.3 cycles of CORT, where only minor deficits were expected and previously reported antidepressant-like effects of Reelin would be more likely (Salk et al., 2017; Brymer et al., 2020; Allen et al., 2022). The rats were weighed 1 day prior to CORT injections and immediately prior to each injection to ensure that accurate doses of CORT were administered. As the animals would be exposed to numerous injections we chose to limit restraint stress during injections. The researcher remained seated throughout and the rats were placed on the researcher after weighing while the syringe was prepared, allowing free exploration. Once the rat settled one hand was placed on top of the rat lightly to prepare the skin for injection, limiting the use of restraint. The researcher remained seated throughout, and the rats were placed on the researcher after weighing while the syringe was prepared, allowing free exploration. Once the rat settled, one hand was placed on top of the rat lightly and prepared the skin for injection, allowing ambulation throughout. Additionally, to mitigate injection stress and promote cooperation, the rats were rewarded with an appetitive stimulus (e.g., a cheerio) after each injection. An *a priori* criterion involved removing animals losing >25% free body weight, although no removal was necessary. A subset of rats received 3 µg of recombinant Reelin (3820-MR-025/CR; R&D Systems; composed of Reelin repeats 3–6, i.e., the central Reelin fragment) dissolved in 0.1% phosphate-buffered saline (PBS) (tail vein; 0.5 mL volume), whereas the rats not receiving Reelin received a vehicle tail vein injection. For the rats exposed to 1.3 cycles, Reelin was administered on day 7 of the second cycle of CORT, and for the rats exposed to 2.6 cycles, Reelin was administered on day 14 of the third cycle of CORT. Reelin was administered approximately 1 h after the final injection of CORT. The dose of Reelin treatment was irrespective of the body mass and was selected based upon past results showing 3 µg of Reelin to be most effective for resolving despair-like behavior [32]. Figure 1 shows an experimental timeline. The groups are as follows: male vehicle/vehicle (MVV); female vehicle/vehicle (FVV); male CORT/vehicle (MCV); female CORT/vehicle (FCV); male CORT/Reelin (MCR); and female CORT/Reelin (FCR). For the 2.6-cycle cohort, n = 6 for each group, and for the female rats exposed to 1.3 cycles, n = 7 for each group.

### Behavioral testing

#### Forced swim test

The FST was conducted 1 day after the final CORT injection and Reelin treatment to evaluate the fast-acting antidepressant-like effects of Reelin. The animals were placed in a Plexiglas tank filled with water to a depth of 30-cm depth (27°C ± 2 °C) and removed after 10 min. The duration of immobility, defined as swimming no more than that required to keep the nose above water, was manually scored to quantify despair-like behavior during the task while blinded to the condition. We considered immobility/body mass to account for differences in buoyancy associated with individual weight differences and evaluated immobility during the final 5 min of the test when immobility tends to be the highest. The Porsolt FST has been the most reliable antidepressant screening tool in rodent research over recent decades, and likewise, no existing antidepressant drug currently available for human consumption fails to resolve despair-like behavior in the FST in rodent models of depression.

#### Open field test

After 24 h of the forced swim test, the animals were introduced to the open field arena (65 cm ×65 cm), which has a light blue floor and black walls of a height of 60 cm. Each rat was placed in a random corner of the apparatus, facing the corner, and the mobility behavior was evaluated across 10 min of exposure to the arena. The activity was recorded by an overhead video camera, and EthoVision (Noldus Information Technology, Wageningen, Netherlands) software was used to automatically calculate the distance traveled, time spent in the center zone (the middle 25 cm × 25 cm of the apparatus), and latency to enter the center. Warm water and soap were used to clean the arena between trials.

### Tissue collection, immunostaining, and histological staining with hematoxylin and eosin

Following the conclusion of behavioral testing, the animals were deeply anaesthetized with 5% isoflurane before transcardial perfusion of 0.9% (w/v) physiological saline (pH of 7.4), followed by the perfusion of 4% paraformaldehyde (pH of 7.4). Spleens and brains were collected and placed in 4% paraformaldehyde for 24 h prior to transferring into 10% (w/v) sucrose in 1× PBS and finally into 30% (w/v) sucrose and 0.1% (w/v) sodium azide in PBS, where the tissue remained until freezing and subsequent cryo-sectioning at 20 µm using a Leica cryostat (CM1850 UV, Germany). Immunohistochemistry was conducted on free-floating coronal sections through the hippocampus for the quantification of Reelin cell counts in the sub-granular zone (SGZ) of the DG. Following initial rinsing with TBS and blocking with 15% (v/v) normal goat serum, 0.5% (v/v) Triton X-100, and 1% (w/v) BSA in 0.1 M TBS, the sections were incubated with the mouse anti-Reelin (Millipore, MAB5364) primary antibody at a concentration of 1:1,000 for 48 h at 4°C. The sections were then rinsed with TBS and incubated for 2 h with a biotinylated horse anti-mouse (1:500, Vector Laboratories, BA-2001) secondary antibody in the blocking solution before being rinsed with TBS and incubated in ABC (1:500, Vector Laboratories) for 1 h. After rinsing with TBS, Reelin immunolabeling was visualized with 0.02% (w/v) DAB and 0.0078% H_2_O_2_. Tissue was rinsed with TBS before being mounted on Superfrost Plus Microscope glass slides and subsequent serial dehydration with ethanol and cover slipping with Permount (Fisher Scientific, SP15-500). For analyzing the percentage of area occupied by white pulp in spleen sections, a portion of each spleen was frozen with liquid nitrogen while being immersed in O.C.T. (Fisher Healthcare, United States) before sectioning. Following slide-mounted serial sectioning, the spleens were stained with hematoxylin and eosin (H&E). Tissue was fixed in 70% ethanol, followed by rinsing with deionized water (dI) before staining with hematoxylin (Gill No. 2, Sigma-Aldrich, Germany) for 1 min, followed by rinsing with dI. Tissue was placed in a bluing reagent of 0.2% sodium bicarbonate (in dI) before placing briefly in acidic ethanol. After another dI rinse, tissues were exposed to alcoholic Eosin Y (Sigma-Aldrich, Germany) for 2 min. Eosin Y staining was followed by ethanol dehydration, exposure to xylene, and cover slipping.

### DG SGZ Reelin-positive cell count and spleen white pulp analysis

A profile count for Reelin-positive cells in the SGZ of the DG was quantified on all coronal sections through the dorsal hippocampus (1 in 12 series) with slide scans using a Zeiss microscope (Imager M2, Zeiss, Germany) at 40×. All sections were counted in the 1 in 12 series containing the dorsal hippocampus, ranging from 6 to 8 sections per subject. The slide scans were loaded in ImageJ Fiji free software (version 1.53c; WS Rasband, National Institute of Health, Bethesda, MD), and a 100 µm × 100-µm grid was overlaid onto the scan. The upper and right grid lines were used as inclusion lines, and the bottom and left grid lines, as exclusion lines; 1 box separated each counted box such that 50% of the SGZ was counted with counts segregated for the dorsal and ventral blades. Cell counting was conducted while being blind to the condition. The spleen sections were imaged using the Zeiss microscope at ×2.5 magnification. The images were converted to binary black and white images by color thresholding in ImageJ using a semi-quantitative method for isolating hematoxylin from images of H&E stains, as described by Reive et al. (2023). An example of binarization of a spleen section is shown alongside both low- and high-powered magnification images of the spleen, where spleen data are reported. White pulp was analyzed while being blind to the subject/condition on three sections per subject, and each section was separated by a minimum of 60 µm. The percentage of the cross-sectional surface area occupied by white pulp was considered from each rat for statistical comparisons.

### Statistics

Statistical analyses were performed using Prism 10 software (version 10.0.2). Group differences in female rats exposed to 1.3 cycles were evaluated with one-way ANOVAs, followed by *post hoc* comparisons using Bonferroni correction for multiple comparisons across all groups. Group differences for rats exposed to 2.6 cycles were evaluated with two-way ANOVAs with the sex and condition as independent variables, followed by *post hoc* testing, where differences were observed with the comparison of each condition within each sex and sex within each condition using Bonferroni correction for multiple comparisons. The results are expressed as the mean ± SEM. Body mass analysis was simplified to evaluate each sex independently with two-way repeated measures (RM) ANOVAs with the day of the procedure and condition (vehicle, CORT/vehicle, and CORT/Reelin) as independent factors using body mass measurements obtained immediately prior to CORT injections from the first and final days of each CORT injection period, although the body mass was evaluated prior to each injection to ensure accurate dosing. *Post hoc* comparisons of each group were compared to those of the other groups on each day of measurement using Bonferroni correction. No removal of outliers was conducted as sample sizes were limited, and the group assignment was randomized. The figures show that group means, SEM, and significance are indicated using asterisks (* = p< .05; ** = p< .01; and *** = p< .001).

## Results

### Minimal weight loss of CORT at 20 mg/kg in males and no weight loss in females

**FIGURE 2:**
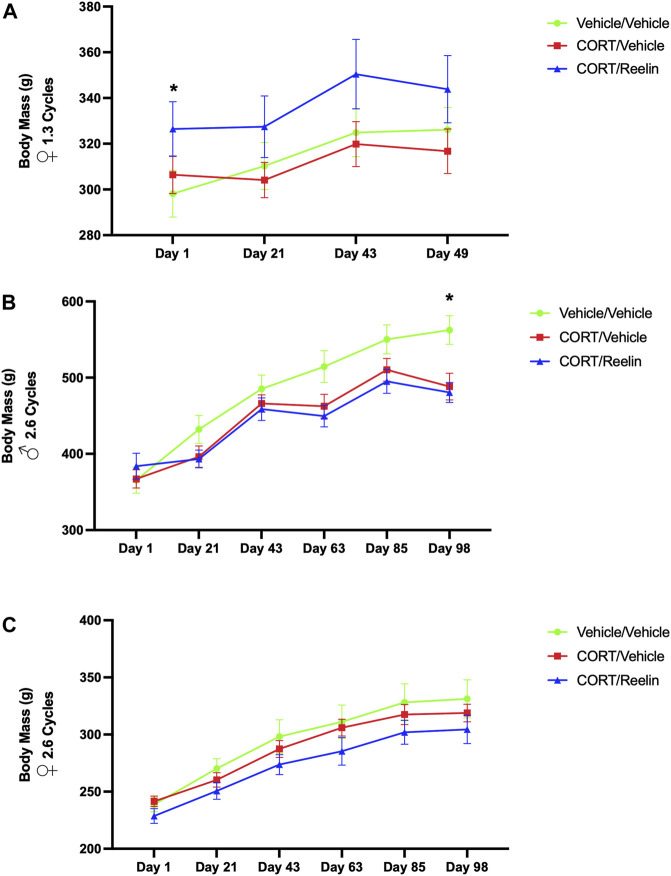
Body mass for 1.3 cycles and 2.6 cycles showing minimal loss of body mass in the cyclic CORT model. **(A)** Body mass of females exposed to 1.3 cycles of CORT with no evidence of weight loss with CORT treatment. The CR group weighed more than the VV group at the start of the experiment despite a random group assignment. **(B)** Body mass for males exposed to 2.6 cycles of CORT with evidence of weight loss in CV and CR groups relative to the VV group on the final day of measurement, day 98 of the experiment. **(C)** Body mass for females exposed to 2.6 cycles of CORT, with no evidence of weight loss due to CORT treatment at any point in the experiment.

The evaluation of body mass (g) of females exposed to 1.3 cycles using two-way RM ANOVA found a significant effect of the condition [F(1.260, 30.24) = 6.743, *p* = 0.010]. *Post hoc* testing revealed that the vehicle/vehicle [M = 298.1; SEM = 10.18] group weighed significantly less than the CORT/Reelin group [M = 326.4; SEM = 11.95] on day 1 of CORT injections [t(6) = 4.489, *p* = .0125] despite the random assignment of groups. No other statistical differences were observed, indicating that 20 mg/kg of CORT was not associated with significant weight loss in females exposed to 1.3 cycles, as shown in Figure 2A.

The results from two-way repeated measures ANOVA for males found a significant interaction between the condition and time [F(10,75) = 15.73, *p* < .0001] and an effect for time [F(2.136, 32.05) = 463.5, *p* < .0001]. *Post hoc* testing revealed a significant reduction in mass on the final day of CORT injections or day 98 of the experiment, with MVV (M = 562.5 g; SEM = 18.825) weighing more than both MCV (M = 488.33 g; SEM = 17.48) (t(9.95) = 2.89, *p* = .049) and MCR (M = 480.5 g; SEM = 13.15) (t(8.94) = 3.57, *p* = .018), as shown in Figure 2B. The results from two-way repeated measures ANOVA of females exposed to 2.6 cycles found a significant effect of time [F(5, 30) = 129.8, *p* < .0001] and condition [F(1.702, 51.06) = 4.987, *p* < .014] but no interaction between time and condition (*p* = .99). No significant differences were observed in *post hoc* testing (p > .647), as shown in Figure 2C. Together, we observed some evidence of weight loss in males treated with CORT after 2.6 cycles but no evidence of significant weight loss in females.

### A single intravenous injection of Reelin resolves immobility in the forced swim test in female rats exposed to the cyclic CORT model and partially recovers immobility in males

**FIGURE 3:**
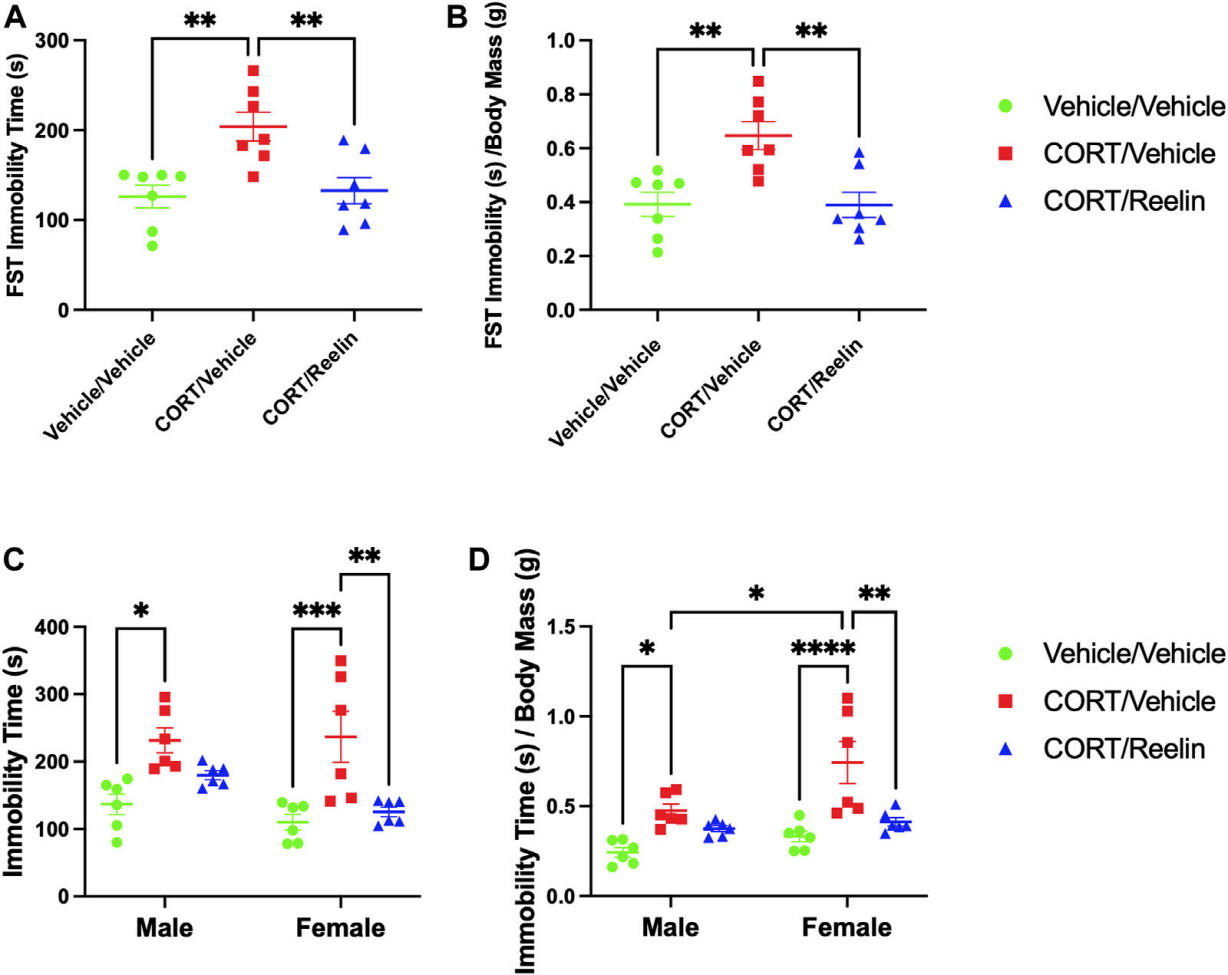
FST results show that CORT elevated FST immobility time and was recovered in females, irrespective of the cohort and partially recovered in males exposed to 2.6 cycles with Reelin. **(A)** FST results of female rats exposed to 1.3 cycles demonstrating significantly increased immobility with CORT that was reversed by Reelin. **(B)** FST results showing that behavioral deficits remained after factoring in individual body mass data. **(C)** FST results showing that CORT induced behavioral despair in both males and females that was recovered with Reelin treatment. **(D)** FST results of rats exposed to 2.6 cycles after factoring in individual body mass, showing that males and females exposed to CORT still show evidence of behavioral despair that was partially recovered with Reelin treatment and that females treated with CORT/vehicle were more immobile than their male counterparts after factoring in individual body mass.

When evaluating behavioral despair in animals exposed to 1.3 cycles of CORT, the results from one-way ANOVA show a significant effect of condition on immobility time [F(2,18) = 8.888, *p* < .002]. *Post hoc* comparisons revealed that rats receiving 1.3 cycles of CORT (M = 204.1; SEM = 15.98) were more immobile than both vehicle/vehicle-treated rats (M = 126.2; SEM = 12.6) [t(18) = 3.801, *p* = .004] and CORT/Reelin-treated rats (M = 132.8; SEM = 14.71) [t (18) = 3.48, *p* = .008], as shown in Figure 3A. No significant difference was observed between vehicle/vehicle-treated rats and CORT/Reelin-treated rats (*p* > .999). When factoring in individual body mass using values calculated from immobility time (s)/body mass (g), the results revealed that the effect of the condition remained [F(2,18) = 9.57, *p* = .002], with *post hoc* tests showing that CORT/vehicle-treated rats (M = 0.647; SEM = 0.052) were still more immobile than vehicle/vehicle-treated rats (M = 0.392; SEM = 0.045) [t (18) = 3.77, *p* = .004], after factoring in body mass, and CORT/Reelin-treated rats (M = 0.39; SEM = 0.047) [t (18) = 3.81, *p* = .004], as shown in Figure 3B. No significant difference was observed between vehicle/vehicle-treated rats and CORT/Reelin-treated rats when factoring in animal mass (*p* > .999). The results show that a single intravenous Reelin injection reversed CORT-induced behavioral despair in animals exposed to 1.3 cycles of CORT.

When analyzing the results from animals exposed to 2.6 cycles, two-way ANOVA of the total immobility time during the FST revealed a significant effect of the condition [*p* < .0001] and no effect of sex (*p* = .119) or interaction between the sex and condition (*p* = .311). *Post hoc* tests revealed increased immobility in MCV (M = 231.553 s; SEM = 18.49) relative to MVV (M = 136.77 s; SEM = 15.16) (t (30) = 3.48, *p* = .014) but no difference between MCR (M = 179.8 s; SEM = 6.55) and either MCV (*p* = .061) or MVV (*p* > .999), as shown in Figure 3C. In females, significant differences were observed between FCV (M = 236.99 s; SEM = 37.7) and both FVV (M = 110.12 s; SEM = 11.59) (t(30) = 4.66, *p* < .0001) and FCR (M = 125.35 s; SEM = 7.09) (t (30) = 4.1, *p* = .003). No difference between FVV and FCR was detected (*p* > .999). When considering individual body mass and FST immobility (as shown in Figure 2B), the results of two-way ANOVA found a significant effect of the condition [F (2,30) = 18.29, *p* < .0001] and sex [F (1,30) = 8.824, *p* = .006]. *Post hoc* tests revealed MCV (M = 0.475; SEM = .04) was more immobile than MVV (M = 0.24; SEM = .03) (t(30) = 3.03, *p* = .045) but not significantly different from MCR (M = 0.38; SEM = .02) (*p* > .999). FCV (M = 0.74; SEM = .12) was more immobile than both FVV (M = 0.33; SEM = .03) (t(30) = 5.37, *p* < .0001) and FCR (M = 0.41; SEM = .02) (t(30) = 4.3, *p* = .002). Between sexes, we found that FCV was significantly more immobile than MCV (t(30) = 3.49, *p* = .014), as shown in Figure 3D. The results demonstrate that Reelin reversed CORT-induced behavioral despair in rats exposed to 2.6 cycles of CORT.

### Lack of behavioral changes in the open field test following cyclic CORT

Evaluation of behavior in the OFT of rats exposed to 1.3 cycles revealed no evidence of behavioral alterations due to chronic stress, with no significant differences found in the total distance (*p* = .66), total duration within the center zone (*p* = .78), or latency to enter the center zone (*p* = .52), as shown in Figures 3A–C.

**FIGURE 4:**
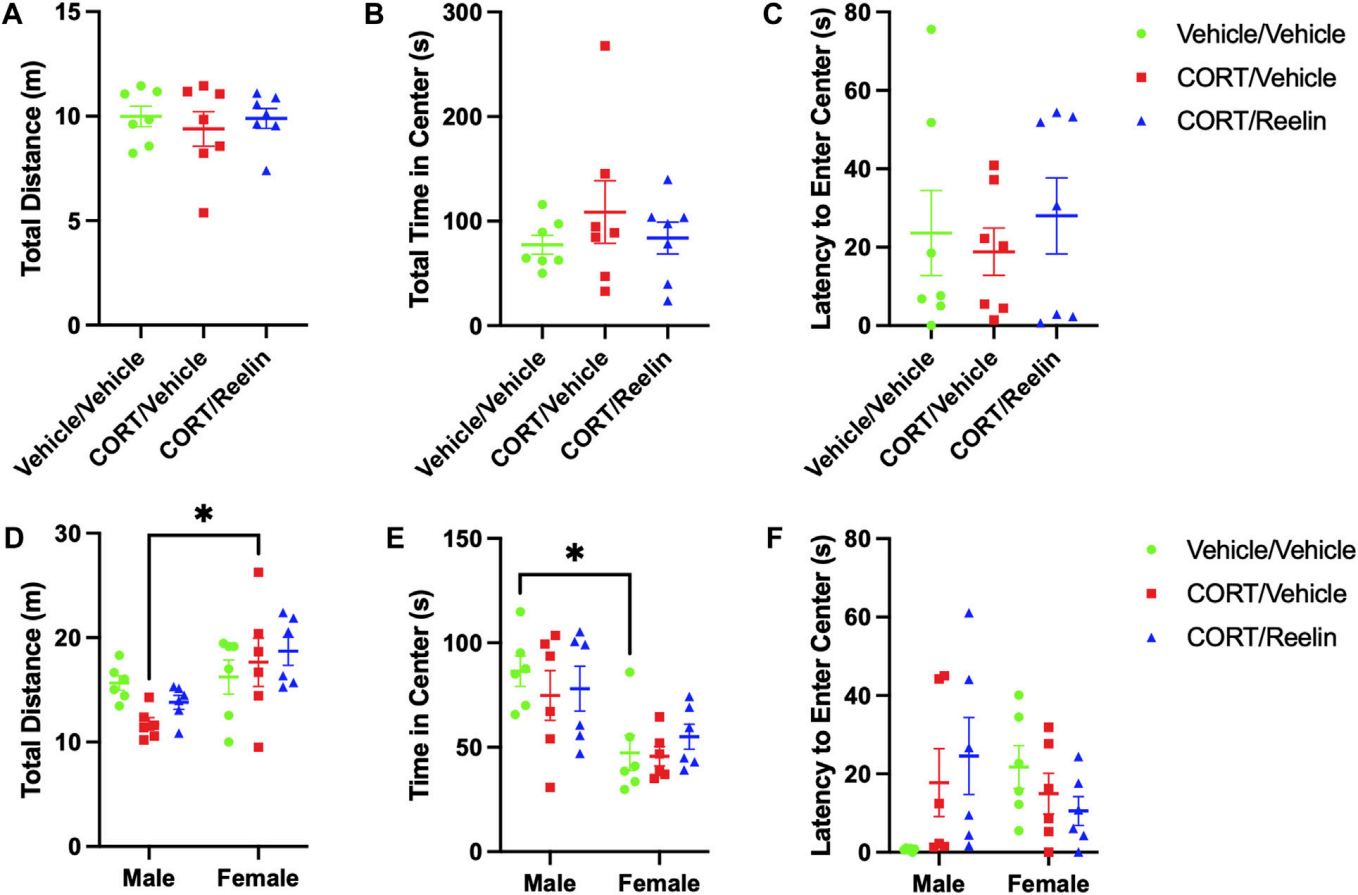
OFT results show that neither CORT nor Reelin altered anxiety-like behavior, and few sex differences were observed. **(A)** Total distance traveled for female rats exposed to 1.3 cycles of CORT shows no alterations in the distance traveled in the OFT. **(B)** Total time in the center region of the OFT showing no alterations in rats exposed to 1.3 cycles. **(C)** Latency to enter the center region in rats exposed to 1.3 cycles, showing no significant alterations due to stress. **(D)** Total distance traveled in the OFT for rats exposed to 2.6 cycles, showing no effect of chronic stress on the distance traveled, but female rats treated with CORT traveled significantly further than males treated with CORT. **(E)** Total time in the center region of the OFT for rats exposed to 2.6 cycles, showing no effect of stress, but female vehicle/vehicle-treated rats spent significantly less time in the center region of the OFT compared to male vehicle/vehicle-treated rats. **(F)** Latency to enter the center zone for rats exposed to 2.6 cycles showing no significant effect of stress or sex differences in the latency to enter the center zone.

When evaluating behavior in the OFT in male and female rats after 2.6 cycles of chronic stress, two-way ANOVA analysis of the results for the total distance revealed a significant effect of sex [F(1,30) = 11.65, *p* = .0019] but no significant effect of the condition (*p* = .491) or interaction (*p* = .132). *Post hoc* tests revealed that FCV (M = 17.7 m; SEM = 2.31) traveled further than MCV (M = 11.75 cm; SEM = 0.60) (t(30) = 3.07, *p* = .041), as shown in Figure 4D. Evaluation of the time spent in the center region of the OFT showed a significant effect of sex [F(1,30) = 18.98, *p* = .0001] but no main effect of the condition (*p* = .684) or an interaction (*p* = .643). *Post hoc* tests revealed that MVV (M = 86.42 s; SEM = 7.28) spent more time in the center region than FVV (M = 47.34; SEM = 8.48) (t(30) = 3.23, *p* = .027), as shown in Figure 4E. Evaluation of the latency to enter the center region of the OFT with two-way ANOVA found a significant interaction between the sex and condition [F(2,30) = 4.001, *p* = .0288]. No main effects were found for sex (*p* = .787) or condition (*p* = .575), and *post hoc* tests revealed no significant group differences in the latency to enter the center region (p > 0.11), as shown in Figure 4F.

### Cycles of CORT reduced DG SGZ Reelin expression and was differentially reversed with intravenous Reelin treatment by the sex and region of the DG

**FIGURE 5:**
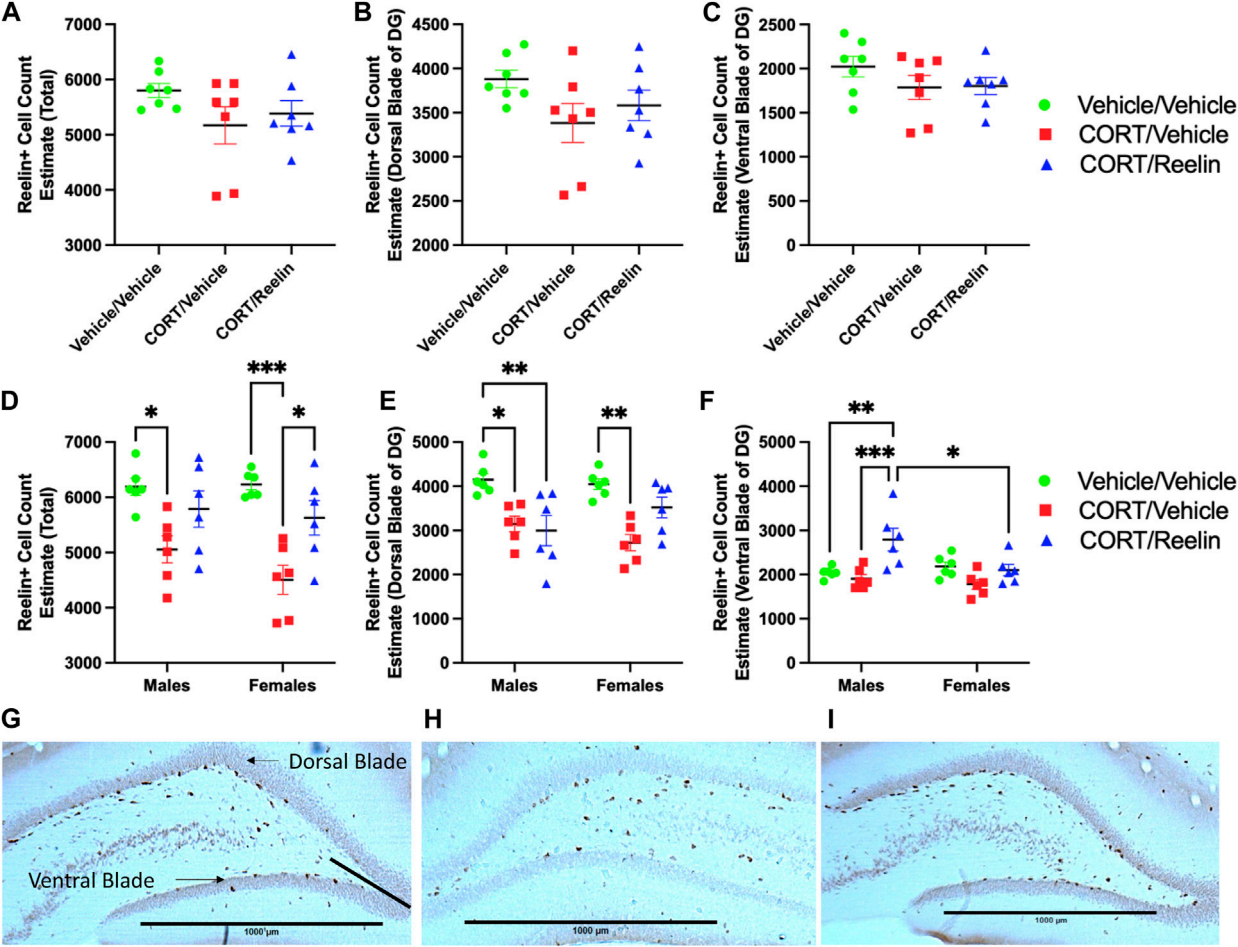
Profile counts for Reelin positive cells shows CORT reduced SGZ Reelin counts after 2.6 cycles, and sex differences to localization of Reelin count alterations after Reelin treatment. Results of Reelin positive cell counts for the SGZ of the DG from rats treated with 1.3 and 2.6 cycles of chronic stress alongside representative images of Reelin immunohistochemistry. **(A)**: Reelin count estimate totals for animals exposed to 1.3 cycles of chronic stress showing no change to Reelin counts after 1.3 cycles of chronic stress. **(B)**: Reelin positive cell count estimates of the dorsal blade of the DG showing no change after 1.3 cycles of chronic stress. **(C)**: Reelin positive cell count estimates of the ventral blade of the DG showing no change after 1.3 cycles of chronic stress. **(D)**: Reelin count estimate totals for animals exposed to 2.6 cycles of chronic stress showing reduced Reelin counts after 2.6 cycles of chronic stress in both males and females that were partially recovered with a single intravenous Reelin injection. **(E)**: Reelin positive cell count estimates of the dorsal blade of the DG showing reduced counts after 2.6 cycles of chronic stress that were partially recovered in females but not males. **(F)**: Reelin positive cell count estimates of the ventral blade of the DG showing no significant reduction to ventral blade counts after 2.6 cycles of chronic stress, however an intravenous injection of Reelin significantly increased Reelin positive cell count estimates for males and not females. **(G)**: Example photomicrograph at 2.5X magnification showing Reelin positive cells in the DG from a rat exposed to vehicle/vehicle injections with demonstration of how dorsal and ventral blades of the DG were differentiated. **(H)**: Photomicrograph at 2.5X magnification showing DG Reelin positive cells from a rat exposed to CORT/vehicle injections. **(I)**: Photomicrograph showing Reelin positive cells in the DG from a rat exposed to CORT/Reelin injections.

To analyze DG SGZ Reelin-positive cell counts, we considered total Reelin + cell count estimates and isolated counts for the dorsal and ventral blades of the DG separately to reveal whether regional differences within the DG occur to Reelin expression after chronic stress and/or Reelin treatment in the cyclic CORT model. One-way ANOVAs used to analyze DG Reelin-positive cell counts in female rats exposed to 1.3 cycles of CORT revealed no significant difference in the total (*p* = .21), dorsal blade (*p* = .15), or ventral blade (*p* = .31) Reelin-positive cell counts, as shown in Figures 5A–C.

When analyzing the total Reelin-positive cell count estimates for rats exposed to 2.6 cycles of treatment, two-way ANOVA revealed a significant effect of the condition [F(2,30) = 17.16, p =< .0001] but no effect of the sex or interaction (p > .27). *Post hoc* analysis revealed a significant reduction in Reelin-positive cell count estimates for males treated with CV (M = 5,056; SEM = 245.28) relative to VV (M = 6,192; SEM = 152.67) (t(30) = 3.34, *p* = .026), as shown in Figure 5D. Similarly, females treated with CV (M = 4,504; SEM = 263.466) showed a significant reduction in Reelin-positive cell count estimates relative to VV (M = 6,232; SEM = 93.706) (t(30) = 4.93, *p* = .0003) and CR (M = 5,628; SEM = 312.35) (t(30) = 3.21, *p* = .029), which is also shown in Figure 5D. No differences were detected between VV and CR for males and females (p > .86). When considering the dorsal blade of the DG, two-way ANOVA revealed a significant effect of the condition [F(2,30) = 16.09, *p* < .0001], but no effect of sex or interaction was observed (p > .089). *Post hoc* testing revealed that in males, VV (M = 4,148; SEM = 137.09) had significantly more Reelin-positive cell count estimates than both CV (M = 3,148; SEM = 173.224) (t(30) = 3.34, *p* = .0199) and CR (M = 2,996; SEM = 342.2) (t(30) = 3.85, *p* = .0051), as shown in Figure 5E. In females, VV (M = 4,048; SEM = 117.85) had higher estimates than CV (M = 2,724; SEM = 183.382) (t(30) = 4.43, *p* = .001), as shown in Figure 5E. No other group differences reached statistical significance (p > 0.1027). The evaluation of Reelin-positive cell count estimates of the DG ventral blade using two-way ANOVA revealed a significant main effect of the condition [F(2,30) = 9.6, *p* = .0006] and a significant interaction [F(2,30) = 4.68, *p* = .017] but no effect of sex (*p* = .0548). *Post hoc* testing revealed that MCR (M = 2,792, SEM = 256.52) estimates were higher than both MVV (M = 2,044; SEM = 50.12) (t(30) = 3.83, *p* = .0055) and MCV (M = 1,908, SEM = 93.93) (t(30) = 4.53, *p* = .0008). This contrasts with the results of females as no significant change in Reelin count estimates was found (p > .3534). The results also showed that MCR (M = 2,792; SEM = 256.52) had significantly higher Reelin cell count estimates than FCR (M = 2,104; SEM = 126.79) (t(30) = 3.52, *p* = .0014), as shown in Figure 5F.

### White pulp atrophy in males exposed to 2.6 cycles of chronic stress recovered with intravenous Reelin treatment

Analysis of the white pulp percentage of the spleen cross-sectional area for rats exposed to 1.3 cycles of chronic stress found no significant change in white pulp with chronic stress (*p* = .895).

**FIGURE 6:**
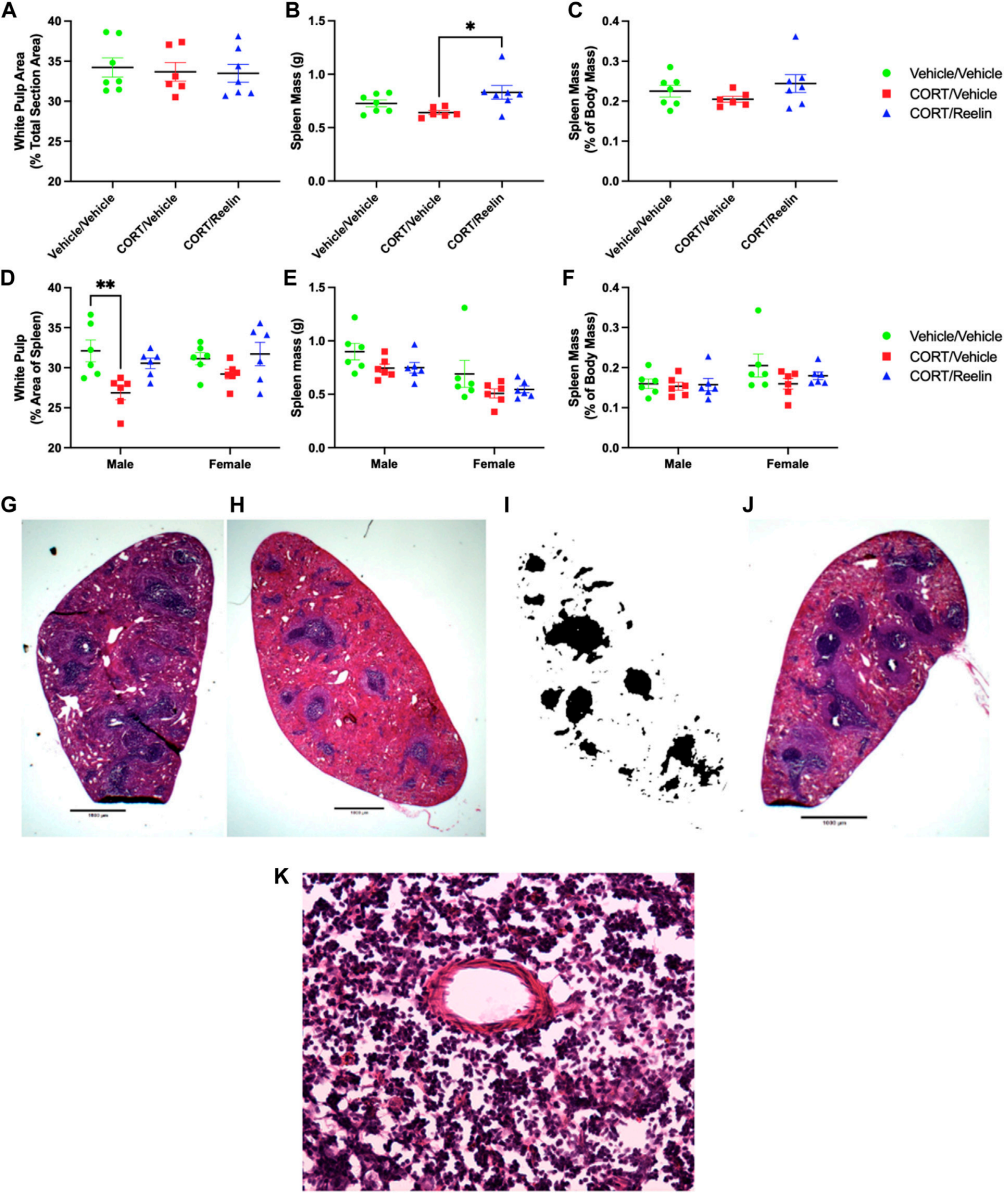
Analysis of spleens shows that Reelin elevated spleen mass in females exposed to 1.3 cycles, and CORT reduced the white pulp area in males only, which was partially recovered with Reelin treatment. **(A)** Percentage of the spleen surface area occupied by white pulp in rats exposed to 1.3 cycles of chronic stress, showing no change in the white pulp percent area. **(B)** Spleen mass of rats exposed to 1.3 cycles of chronic stress, showing no significant deficit in spleen mass with chronic stress, but animals exposed to both chronic stress and Reelin injections had heavier spleens than rats exposed to chronic stress alone. **(C)** Spleen mass expressed as the percentage of body mass, showing no significant differences in rats exposed to 1.3 cycles of chronic stress. **(D)** Percentage of the spleen surface area occupied by white pulp in rats exposed to 2.6 cycles of chronic stress, showing a deficit in the white pulp area in males but not females, which was partially restored after Reelin treatment. **(E)** Spleen mass in animals exposed to 2.6 cycles of chronic stress treatment, showing no significant difference in the mass between treatments. **(F)** Spleen mass expressed as the percentage of total body mass for animals exposed to 2.6 cycles of chronic stress treatment, showing no significant change in the spleen mass relative to body mass between any treatment groups. **(G)** Photomicrograph at ×1 magnification demonstrating the H&E staining of a section obtained from a male rat exposed to vehicle/vehicle injections. **(H)** Photomicrograph at ×1 magnification of the spleen from a CORT/vehicle-treated male rat after H&E staining. **(I)** Binarization of Image 8H demonstrating how H&E-stained images were converted to black and white for quantifying white pulp. **(J)** Photomicrograph at ×1 magnification of the spleen from a CORT/Reelin-treated male rat after H&E staining. **(K)** Photomicrograph at ×40 magnification of an H&E-stained spleen section within the white pulp region from a vehicle-treated rat exposed to 2.6 cycles of injections that shows the presence of artifacts, likely created during cryosectioning.

Analysis of spleen mass for rats exposed to 1.3 cycles of chronic stress using one-way ANOVA found a significant group difference [F(2,17) = 4.376, *p* = .0293], with *post hoc* tests revealing that the spleen mass was lower in CV (M = 0.642 g; SEM = 0.019) than that in CR (M = 0.83 g; SEM = 0.0638) (t(17) = 2.944, *p* = .0272), but no other significant differences were found (p > .339), as shown in Figure 6B. When factoring in individual body mass, we see no significant change in the spleen mass with treatment (*p* = 0.282), as shown in Figure 6C.

Analysis of the percentage of the spleen cross-sectional surface area occupied by white pulp using two-way ANOVA found a significant effect of the condition [F(2,30) = 7.447, *p* = .0024], and *post hoc* testing revealed that the male CV group (M = 26.855%; SEM = 0.87) had significantly less area occupied by white pulp compared to the male VV group (M = 32.1%; SEM = 1.36) (t(30) = 3.684, *p* = .0081), but no other significant differences were observed (p > .129). The results shown in Figure 6D show that Reelin partially recovered the white pulp area in males, and although a similar pattern was observed in females, the differences were not statistically significant. Two-way ANOVA of spleen mass data on rats exposed to 2.6 cycles revealed a significant main effect of sex [F(1,30) = 14.63, *p* = .0006] and condition [F(2,30) = 3.55, *p* = .0414], but *post hoc* testing revealed no significant differences between conditions for males and females (p > .198), as shown in Figure 6E. When analyzing spleen mass represented as the percentage of individual animal mass, two-way ANOVA revealed no significant effect of the condition, sex, or interaction (p > .0702), indicating that all groups had a similar proportion of body mass occupied by the spleen, as shown in Figure 6F. It should be considered that body mass of the live animal was calculated on the final day of the experiment, whereas the mass for spleens was obtained after perfusions, which likely impacts the density and, therefore, mass of spleens, and presumably, the extent to which spleen mass is affected varies from animal to animal.

## Discussion

Evaluating novel antidepressants like Reelin in a model of recurring depressive episodes helps determine whether the treatment efficacy is impacted by the duration of illness, which varies drastically across individuals seeking treatment for depression. Here, we found that both male and female rats treated with Reelin after chronic stress recovered immobility in the FST relative to those exposed to chronic stress not receiving Reelin. The results suggest that a single dose of Reelin maintains antidepressant-like effects in male and female rats exposed to multiple bouts of chronic stress, as shown in Figure 3. The findings replicate past reports showing that Reelin resolves FST immobility after prolonged exposure to chronic stress in the form of repeated CORT injections (Brymer et al., 2020; Allen et al., 2022). FST immobility was partially recovered in males exposed to 2.6 cycles of CORT, whereas female rats had more complete recovery of immobility behavior in the FST after 2.6 cycles, suggesting that dose refinement may be important for future studies, particularly in males.

SGZ Reelin-positive cell counts were depleted in both males and females after 2.6 cycles of CORT exposure, replicating a previous assessment of SGZ Reelin in male rats, following cyclic CORT (Lebedeva et al., 2020). We also found that intravenous Reelin normalized estimates after cyclic CORT (as shown in Figure 5D). These results replicate those of previous studies evaluating the antidepressant-like effects of Reelin after 21 days of CORT, in which Reelin administered intravenously restored SGZ Reelin cell counts (Allen et al., 2022; Johnston et al., 2023). Sex differences were observed when segregating counts into the dorsal and ventral blades of the DG, with dorsal blade SGZ counts restored with Reelin in females but not males, whereas cell counts were elevated beyond normal levels after Reelin treatment in the ventral blade of male rats (Figure 5F). Although there is limited research evaluating regional differences in Reelin expression in the DG, this could impact neurogenesis in the DG as Reelin was previously shown to normalize CORT-induced neurogenesis alterations, and past research found altered levels of neurogenesis in the DG across these regions (Choi et al., 2003; Uemura et al., 2021; Allen et al., 2022). Sex differences in the localization of Reelin after treatment could impact neurogenesis as sex differences to neurogenesis have also been shown after chronic stress (Hillerer et al., 2013). It does not appear that these sex differences are important for the antidepressant-like effects of Reelin treatment as Reelin reversed FST immobility in both males and females.

In contrast to the cohort exposed to 2.6 cycles, we see no deficits in SGZ Reelin counts after 1.3 cycles. Previous results of the cyclic CORT model found that Reelin counts were not normalized in males after a stress-free period of 21 days after the first 21 days of CORT, although females were not previously included (Lebedeva et al., 2020). The absence of deficits in females after 1.3 cycles suggests that SGZ Reelin may be naturally restored in females in the absence of treatment with the removal of stressors. As we expected deficits to be less susceptible to recovery in the third cycle of CORT, we evaluated the antidepressant-like effects of Reelin in both sexes only after 2.6 cycles. We decided to evaluate females in the 1.3-cycle cohort as depressive disorders predominantly affect females, and a previous assessment of the cyclic CORT model only involved male rats (reviewed by Mohammadi et al., 2023; Salk et al., 2017; Lebedeva et al., 2020).

The spleen cross-sectional surface area occupied by white pulp following CORT was reduced in males but not females, and deficits were restored with Reelin treatment (Figure 6D). This result suggests that Reelin reversed immune dysfunction caused by chronic stress, as previously reported (Reive et al., 2023). Although longer durations of chronic stress are associated with more severe atrophy of white pulp integrity, the reduced dose of CORT used here (20 mg/kg rather than 40 mg/kg) could explain the absence of previously observed deficits in females (Hernandez et al., 2013; Reive et al., 2023). Although the roles of Reelin in regulating inflammation are poorly understood at present, it was recently shown that Reelin is elevated in individuals with mild and severe symptoms of COVID-19, and experimentally preventing Reelin upregulation led to less severe COVID-19 symptoms, following COVID-19 infection (Calvier et al., 2023). The role of Reelin in inflammation was recently reviewed (Alexander et al., 2023); however, the various disorders marked by both low Reelin expression and elevated markers of inflammation, such as major depression, schizophrenia, and Alzheimer’s disease, were not considered (Fatemi et al., 2000; Miller et al., 2009; Herring et al., 2012; Muller et al., 2015; Novoa et al., 2022). The replication of an anti-inflammatory-like effect in the spleen, following Reelin treatment, in chronically stressed animals suggests that restoring Reelin homeostasis can regulate chronic stress-induced inflammation and that Reelin is not strictly pro-inflammatory, as previously described (Alexander et al., 2023; Calvier et al., 2023).

In the OFT, which is used to evaluate anxiety-like behavior, we observe several sex differences but no effect of chronic stress, suggesting that cyclic CORT did not induce anxiety-like behavior (Seibenhener and Wooten, 2015). In terms of sex differences, males treated with CORT traveled further than CORT-treated females (Figure 4D), and males treated with a vehicle/vehicle spent significantly more time in the center zone than females treated with a vehicle/vehicle (Figure 4E), suggesting that female rats showed more anxiety-like behavior than males. Considering OFT mobility in the interpretation of mobility differences during the FST, the mean distance traveled in the OFT is not significantly different between males and females after CORT treatment (Figures 4A&D). Multiple observations of CORT increasing FST immobility, here and in previous studies, in conjunction with normal levels of mobility during the OFT, lead us to conclude that reduced mobility in the FST is not related to general mobility reduction and, instead, is related to despair-like behavior evoked by chronic stress (Johnson et al., 2006; Brymer et al., 2020; Allen et al., 2022; Johnston et al., 2023).

We observe less variability in despair-like behavior in female rats exposed to 2.6 cycles of CORT receiving Reelin than those exposed to 1.3 cycles. An unaccounted-for effect of estrus cycles may be involved as Reelin is regulated by the estrus cycle (Meseke et al., 2018). Potentially, a lower dose of Reelin could be sufficient for rats in the pro-estrus phase exposed to 1.3 cycles of chronic stress as Reelin counts were not reduced in the SGZ after 1.3 cycles, as shown in Figures 5A–C. Although we lack an understanding of whether Reelin expression in the brain is affected by the estrus cycles and peripheral Reelin, multiple lines of evidence support elevating Reelin levels in the periphery through the intravenous administration of recombinant Reelin to restore Reelin levels in the brain (Allen et al., 2022; Johnston et al., 2023). Despite choosing a single dose of Reelin, we replicated past findings of antidepressant-like effects of Reelin treatment (Brymer et al., 2020; Allen et al., 2022; Johnston et al., 2023).

One control group absent from this study is a vehicle-treated group receiving Reelin. The impact of 3 µg of Reelin on vehicle-treated rats has been considered in a previous study for each measurement evaluated (Allen et al., 2022; Reive et al., 2023). As this group is not necessary to evaluate the antidepressant-like effects of Reelin after multiple cycles of chronic stress, there is limited benefit to including this control group here. Reliance on the FST to evaluate despair-like behavior is a limitation to this research; however, the FST has been the most effective screening tool for antidepressants over recent decades, and multiple observations that Reelin restores FST immobility suggest that Reelin has potential therapeutic value for treating depression (Armanio, 2021; Allen et al., 2022; Johnston et al., 2023). Social isolation stress is another limitation to this study as both control and experimental groups are exposed to a significant duration of social isolation, which has been used previously to evoke a despair-like phenotype, and with much shorter durations (Djordjevic et al., 2012). Future studies with the cyclic CORT model could consider re-establishing partner housing during the recovery period to limit social isolation stress. Although the restraint was intentionally limited during CORT injections, a restraint was required for the tail vein injections, representing an additional stressor. However, past studies from our laboratory have shown that restraint stress does not cause significant changes in immobility in the FST compared to CORT (Gregus et al., 2005; Johnson et al., 2006), and the restraint does not alter Reelin expression in the DG SGZ in contrast to repeated CORT injections (Lussier et al., 2009). Profile counts are another limitation, and Reelin counts may be underestimated as slide scans were obtained at a single *Z*-axis (Mura et al., 2004). A ×40 magnification image of the white pulp region from a male vehicle/vehicle treated rat shown in Figure 6K shows the presence of several artifacts. This was included to demonstrate a limitation with the spleen analysis. These artifacts were observed in all sections observed at a higher magnification, irrespective of the condition, and are likely a result of cryosectioning, as reported previously (Accart et al., 2014). The combination of higher-magnification and lower-magnification images demonstrates that although the fine structure of the spleen was disrupted, the gross morphology of the spleen remained intact, warranting the use of low-magnification analysis of the spleen to analyze white pulp morphology.

This study supports the use of the cyclic CORT model for evaluating novel therapeutics for depressive disorders in a model of recurring depressive episodes. This is important because humans experiencing depressive disorders commonly experience multiple depressive episodes, which can be separated by months or years, and this can impact antidepressant treatment resistance (Dudek et al., 2010). Previous research with the model demonstrated various behavioral and neurobiological changes, but no evaluation of therapeutics had been conducted within the model. The evaluation of novel therapeutics in a model of multiple depressive episodes may improve the translation of antidepressant efficacy in pre-clinical animal models to humans; however, further validation of this model of recurring depressive episodes should be conducted using conventional antidepressants, such as SSRIs, and fast-acting antidepressants, such as ketamine. Future directions for the evaluation of Reelin as an antidepressant include evaluating the antidepressant-like effects in additional animal models (such as chronic unpredictable mild stress, repeated restraint stress, social instability stress, and early life stress). Determining the toxicity and bioavailability of Reelin treatment through various routes of administration and the exploration of potential negative effects of altering Reelin levels with recombinant Reelin are important prior to conducting trials in humans. It would also be valuable to evaluate whether treatment with Reelin reduces susceptibility to develop depressive symptoms, heart disease, and cognitive dysfunction in the context of healthy aging and in the context of future stress exposure(s).

## Conclusion

The results shown here demonstrate that Reelin has antidepressant-like effects in a rodent model of recurring depression. DG SGZ Reelin counts were depleted in the cyclic CORT model but reversed by a single tail vein injection of Reelin. Reelin treatment also resolved CORT-induced white pulp atrophy in males, as previously shown in the 21-day CORT model (Reive et al., 2023). This work supports the continuation of research evaluating Reelin as an antidepressant.

## Data Availability

The original contributions presented in the study are included in the article/Supplementary Material; further inquiries can be directed to the corresponding author.
